# Immunohistochemical expression of four different stem cell markers in prostate cancer: High expression of NANOG in conjunction with hypoxia-inducible factor-1α expression is involved in prostate epithelial malignancy

**DOI:** 10.3892/ol.2014.2274

**Published:** 2014-06-23

**Authors:** KATSUHITO MIYAZAWA, TAKUJI TANAKA, DAN NAKAI, NOBUYO MORITA, KOJI SUZUKI

**Affiliations:** 1 Department of Urology, Kanazawa Medical University, Uchinada, Ishikawa 920-0293, Japan; 2 Department of Diagnositic Pathology and Research Center of Diagnostic Pathology, Gifu Municipal Hospital, Gifu, Gifu 500-8513, Japan

**Keywords:** cancer stem cell, NANOG, OCT4, prostate cancer, hypoxia-inducible factor-1α

## Abstract

Cancer stem cells (CSCs) have been identified in a variety of cancer types, including prostate cancer. The aim of the present study was to evaluate the immunohistochemical expression of NANOG, octamer 4 (OCT4), cluster of differentiation 133 (CD133) and NESTIN, which are all CSC markers, and assess their function in prostate carcinogenesis. A total of 114 patients were referred to the Kanazawa Medical University Hospital (Uchinada, Japan) having presented with elevated serum prostate-specific antigen levels and/or abnormal digital rectal examinations, and underwent transrectal ultrasound sonography guided eight core biopsies. The prostate pathological specimens were re-evaluated for selection in this study. When specimens were diagnosed as prostate cancer, immunohistochemical analysis of the four different stem cell markers (NANOG, OCT4, CD133 and NESTIN) and hypoxia-inducible factor (HIF)-1α was performed. Prostate cancer was found in 38 cases (33.3%), while the other patients had benign prostate hyperplasia with prostatitis. All prostate cancers were histopathologically identified as adenocarcinomas of various grades, and cancer cells and intraepithelial neoplasia (high grade) were immunohistochemically shown to express NANOG and OCT4, but not CD133 and NESTIN. The intensity of NANOG expression was much greater than that of OCT4, and the positivity and intensity of the four stem cell markers, including NANOG, were elevated with high Gleason scores. A significant correlation was observed between the NANOG- and HIF-1α-positive regions. The CSC markers, in particular OCT4 and NANOG, were immunohistochemically expressed in prostate cancers. Furthermore, HIF-1α expression may affect NANOG and/or OCT4 expression. The findings of the current study suggested that NANOG expression may be a biomarker for the diagnosis of prostate cancer, and the coexpression of NANOG and HIF-1α may be involved in prostate carcinogenesis.

## Introduction

Prostate cancer is the second leading type of malignancy in males in North America with an estimated 186,320 new cases and 28,660 mortalities reported in 2008 (1). The number of patients with prostate cancer has also been increasing in Japan (2). Alterations in nuclear morphometry, gene and protein expression, gene promoter methylation and angiogenesis are known to be involved in prostate carcinogenesis and contribute to field cancerization in the prostate (3).

Our understanding of carcinogenesis has been enhanced by the recently revived cancer stem cell (CSC) theory. CSCs have been reported in multiple solid tumors in different tissues, including the prostate (4–6). CSCs are endowed with high tumorigenic capacity and may drive tumor formation, maintain tumor homeostasis and mediate tumor metastasis. A number of primary non-malignant and malignant tumor-derived human prostate epithelial cell lines have been developed using a retroviral vector encoding human telomerase reverse transcriptase. These cell lines exhibit the characteristics of stem cells and express embryonic stem (ES) cell markers, such as NANOG, octamer 4 (OCT4) and SRY-box 2 (Sox-2), as well as the early progenitor cell markers, cluster of differentiation 133 (CD133), CD44 and NESTIN (7,8).

The multipotent stem cell marker NANOG was identified in 2003 (9,10). NANOG is specifically expressed in the human embryonic pluripotent stem cells of embryos prior to or following implantation, primordial germ cells, ES cells cultured *in vitro*, embryonic germ cells and embryonic carcinoma cells, and functions in the promotion of cell proliferation. NANOG is expressed in dysgerminoma and embryonic carcinoma, but not in immature teratoma, endodermal sinus tumors or choriocarcinoma (11). NANOG can be used to distinguish between germ cell tumors and non-germ cell tumors (11). NANOG has also been found to be a sensitive and specific marker of metastatic germ cell tumors (11,12). With regard to prostate cancer, several studies have recently suggested the positive reaction of adenocarcinoma (ADC) cells against NANOG (13,14). Therefore, NANOG is an emerging focus in developmental biology, due to its importance in the maintenance of self-renewal and multipotential capacity in a variety of malignancies, including prostate cancer. Octamer 4 (OCT4) belongs to the family of Pit-Oct-Unc-domain transcription factors and has been found in ES and germ cells (15). A number of reports have shown that OCT4 is pivotal in maintaining the self-renewal and pluripotency of ES cells (16). Recently, it has also been shown that cancer cells expressing OCT4 and Sox2 may be crucial in cancer development (17). The two genes, Sox2 and OCT4, are part of an important gene regulatory network, and are essential for embryogenesis and the pluripotency and self-renewal of cells (16). Previous studies have also suggested that certain cancers, including prostate cancer (14,18), express Sox2 and OCT4 simultaneously (19,20), and their expression has been associated with the differentiation of tumors (21). These two genes are significant for cancer cell survival. CD133 is a transmembrane glycoprotein that is originally expressed in a subset of stem cells in the hematopoietic system as well as in the solid tumors of other tissues (22), including the prostate (23). CD133-positive cancer cells have cancer stem/progenitor cells that exhibit resistance to cancer therapies (including radiation and chemotherapy), a greater invasion ability and metastasis in various malignancies. Thus, the utility of CD133 expression as a prognostic marker has been suggested (22), as well as in the prostate (23). NESTIN is an intermediate filament protein that is known to be important as a neural stem cell marker (24). However, the expression of NESTIN has recently been reported to be associated with the proliferation of progenitor cell populations within neoplasms (25). In addition, the upregulation of NESTIN has been found to closely correlate with the malignancy and metastasis of a variety of malignancies (25), including prostate cancer (26).

The expression of NANOG, OCT4, Sox, NESTIN and CD44 has been observed in human prostate ADC cells (7), which suggests the importance of cancer stem and progenitor cells in prostate carcinogenesis. However, OCT4A-expressing cells have rarely been identified in human benign and malignant prostate glands (27). The number of OCT4A-expressing cells has been shown to increase in prostate ADC with high Gleason scores (27). OCT4A-expressing cancer cells have also been shown to coexpress Sox2, an ES cell marker, but did not express other putative stem cell markers, such as NANOG and CD133 (27). The neuroendocrine differentiation markers, chromogranin A and synaptophysin, are also coexpressed by the majority of OCT4A-expressing cells (27). Thus, discrepancies exist in reports investigating the role and expression of certain stem and progenitor cell markers in prostate cancer cells.

In the current study, in order to determine whether certain stem cell markers may be used for the diagnosis of prostate cancer, the immunohistochemical expression of NANOG, OCT4, CD133 and NESTIN, which are well-known stem cell markers, were investigated in 38 cases from a total of 114 biopsy specimens obtained from Japanese patients with prostate cancer between January 2011 and December 2011. In addition, the correlation between the expression of these stem cell markers in prostate cancer and non-cancerous tissues was evaluated. Hypoxia has been associated with an aggressive course and poor clinical outcome of cancer (28,29); low oxygen may promote the self-renewal of CSCs (14,30–32). Therefore, the immunohistochemical expression of hypoxia-inducible factor (HIF)-1α was also examined.

## Materials and methods

### Study samples

Between October 2010 and September 2011, a total of 114 patients with elevated serum prostate-specific antigen levels of >4 ng/ml and/or abnormal digital rectal examinations were referred to the Kanazawa Medical University Hospital (Uchinada, Japan) and underwent transrectal ultrasound sonography-guided eight-core biopsies. Histopathological diagnosis was re-evaluated by a certified pathologist on hematoxylin and eosin-stained sections from the biopsy samples. Prior to this study, written informed consent was obtained from all patients. The study was approved by the Ethics Committee of Kanazawa Medical University (Uchinada, Japan), and the Declaration of Helsinki regarding the use of human tissue was strictly followed.

### Immunohistochemistry

Serial sections, 4 μm in thickness, prepared from formalin-fixed, paraffin-embedded specimens, were available for immunohistochemical analysis. Sections were deparaffinized and rehydrated following standard methods. Briefly, the sections were deparaffinized three times with xylene for 5 mins, and rehydrated in graded ethanol (80–100%) for 5 mins. A microwave antigen retrieval procedure was performed for 20 min in citrate buffer (pH 6.0) and hydrogen peroxide was used to block non-specific peroxidase reactions. Following washing with phosphate-buffered saline (PBS, pH 7.4), sections were incubated with rabbit polyclonal anti-human NANOG (ab21624; 1:30 dilution; Abcam, Cambridge, MA, USA), OCT4 (ab18976; 1:100 dilution; Abcam), CD133 (ab19898; 1:200 dilution; Abcam) and NESTIN (ab93666; 1/120 dilution; Abcam), as well as mouse monoclonal anti-human HIF-1α (ab10625; 1:200 dilution; Abcam). Following washing three times with PBS, sections were incubated at 37°C with biotin-conjugated goat anti-rabbit polyclonal antibody (ab6720; Abcam) for 20 min. Visualization was achieved by incubation with diaminobenzidine for 10 min and slides were counterstained with Mayer hematoxylin. Following hydration in graded alcohol and clearing with xylene, the slides were mounted with neutral gum. Seminomas obtained from testicular cancer specimens of two patients (Kanazawa Medical University Hospital) who had undergone surgical resection, which had been confirmed to overexpress NANOG and OCT4, were selected as the appropriate positive controls (33), and paraffin-embedded Caco-2 cells (cat. no. CRL-2102; American Type Culture Collection, Manassas, VA, USA) and endothelial cells in ADC obtained from colorectal cancer specimens of two patients (Kanazawa Medical University Hospital) who had undergone surgical resection were used as internal positive controls for CD133 and NESTIN (34,35). Negative controls were prepared by incubating samples without the primary antibody. The intensity of immunoreactivity against all the primary antibodies used was assessed using a microscope (Olympus BX41; Olympus Optical, Tokyo, Japan). Indices were determined by counting the number of positive nuclei among ≥300 cells in high-power fields, and were indicated as percentages. Positive cells were evaluated for their intensity of immunoreactivity on a 0 or 3+ scale. The overall intensity of the staining reaction was scored as follows: 0, no immunoreactivity and no positive cells; 1 (+/−), weak expression in <50% cells; 2 (+), moderate expression in ≥50% cells; 3 (++), moderate to strong expression in 51–75% cells; and 4 (+++), strong and diffuse expression in >76% cells. Slides were reviewed by one pathologist blinded to the clinical data.

### Statistical analysis

Incidences among the groups were compared using a two-tailed unpaired t-test and Bonferroni multiple comparison test (GraphPad InStat version 3.05; GraphPad Software, San Diego, CA, USA). P<0.05 was considered to indicate a statistically significant difference between the groups.

## Results

### General observations

**Figure 1:**
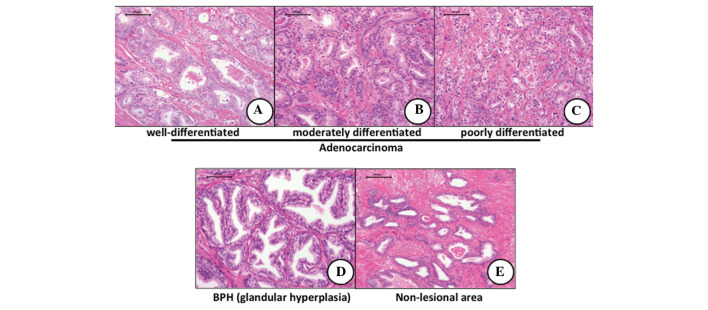
Histopathology of biopsy specimen. Prostate biopsy specimen: (A–C) Adenocarcinoma (magnification, ×100), (D) benign prostate (glandular) hyperplasia and (E) non-lesional area (magnification, ×200). Gleason scores: (A) 2+2; (B) 3+3 and (C) 4+4 (hematoxylin and eosin stain; scale bars, 100 μm).

Prostate cancer was found in 38 (33.3%) of 114 males who underwent eight core biopsy and were divided into two subgroups according to the following Gleason scores: 30 cases with <6 (3+3) and eight cases with >7 (3+4), as shown in Fig. 1A–C. Other specimens were diagnosed as benign prostate hyperplasia with marginal prostatitis (Fig. 1D) or normal prostate glands (Fig. 1E).

### Immunohistochemical findings

**Figure 2:**
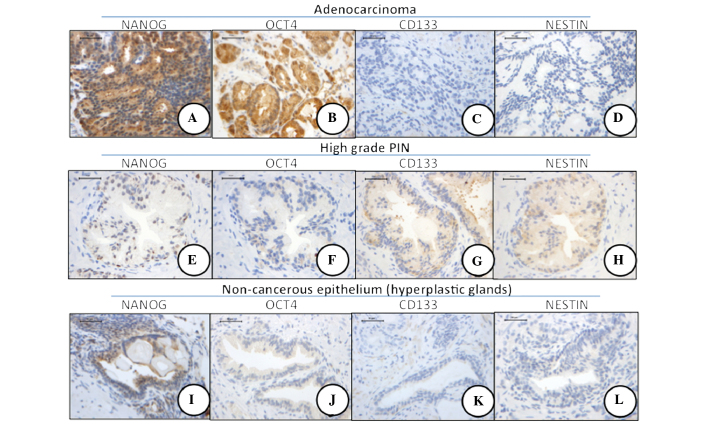
Immunohistochemistry of four stem cell markers in prostate adenocarcinoma and non-cancerous epithelium. NANOG and OCT4 proteins were mainly localized in the nucleus and cytoplasm of the tumor cells of prostate cancer. (A) NANOG expression was strong in cancer cell nuclei, while (E) OCT4 expression was weak (magnification, ×200; scale bars, 50 μm). OCT4, octamer 4.

Of the four stem cell markers, ADC cells in all specimens of the 38 cases of prostate ADC were found to positively express the NANOG (Fig. 2A) and OCT4 (Fig. 2B) proteins. However, the immunohistochemical expression of CD133 (Fig. 2C) and NESTIN (Fig. 2D) was extremely weak or absent in the cancer cells of prostate ADC and those of non-cancerous cells. High-grade prostate intraepithelial neoplasia (PIN) was positive for NANOG (Fig. 2E) and OCT4 (Fig. 2F); however, the number of positive cells was fewer than that of prostate cancer. The majority of hyperplastic glands were negative for NANOG (Fig. 2I) and OCT4 (Fig. 2J) staining. The cells of hyperplastic glands were completely negative for CD133 (Fig. 2K) and NESTIN (Fig. 2L). Positive reactions for NANOG and OCT4 were predominantly localized in the nuclei of cancer cells and the cell nuclei of PIN. The staining intensity of NANOG was stronger than that of OCT4.

**Figure 3:**
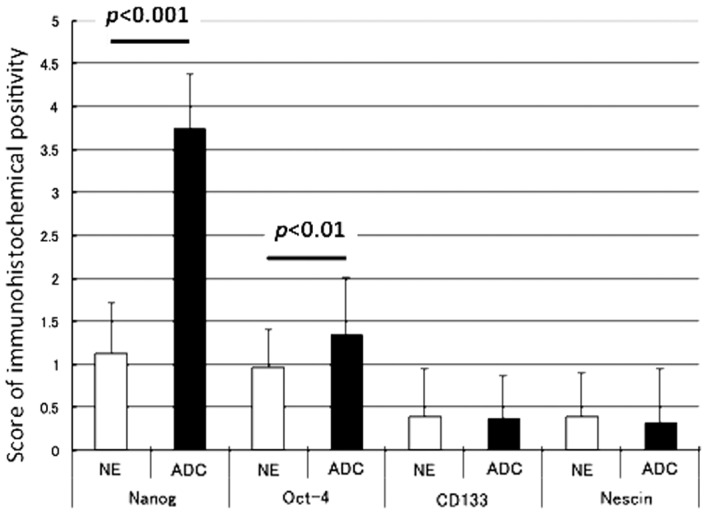
Immunohistochemical expression scores of four stem cell markers in prostate ADC and NE. The scores of NANOG and OCT4 in ADC were significantly higher than that in the NE (P<0.001 and P<0.01, respectively). ADC, adenocarcinoma; NE, non-cancerous epithelium; OCT4, octamer 4.

**Figure 4:**
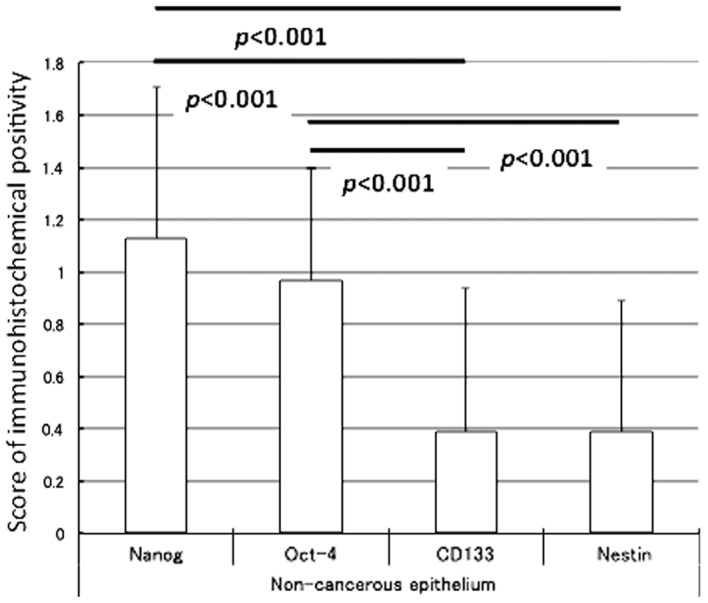
Immunohistochemical expression scores of four stem cell markers in non-cancerous epithelium. The scores of NANOG and OCT4 were higher than those of CD133 and NESTIN (P<0.001). No significant difference was identified between the scores of NANOG and OCT4. OCT4, octamer 4; CD133, cluster of differentiation 133.

**Figure 5:**
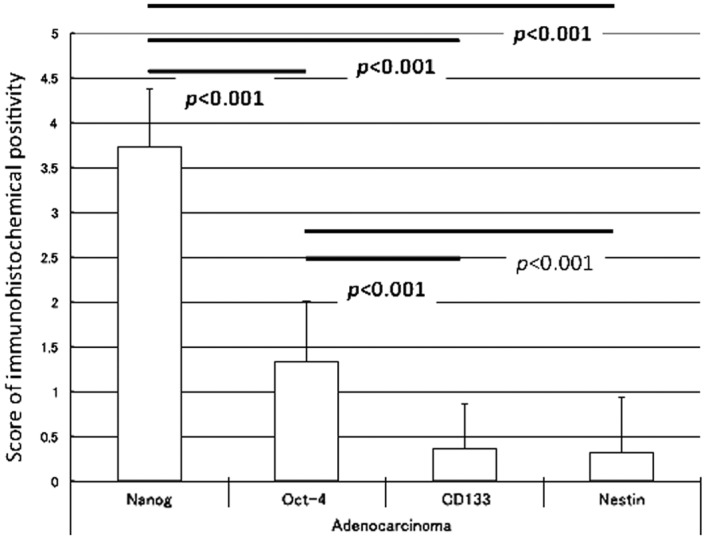
Immunohistochemical expression scores of four stem cell markers in prostate adenocarcinoma. The score of NANOG was the highest among the four stem cell markers, and the value was significantly greater than that of OCT4 (P<0.001), CD133 (P<0.001) and NESTIN (P<0.001). The score of OCT4 was significantly higher than that of CD133 (P<0.001) and nestin (P<0.001). OCT4, octamer 4; CD133, cluster of differentiation 133.

Fig. 3 shows the scorings for the immunohistochemical expression of the four different stem cell markers in prostate cancer and non-cancerous cells. The immunoreactivities of NANOG (P<0.001) and OCT4 (P<0.01) in prostate cancer was significantly greater than those in the non-cancerous cells. No significant differences were identified between the immunoreactivities of CD133 and NESTIN in the prostate cancer and non-cancerous cells. Based on the detailed analysis, the scoring data for the four different stem cell markers in the non-cancerous and prostate cancer cells are also illustrated in Figs. 4 and 5, respectively. The immunohistochemical intensity of prostate cancer was weakest for NANOG followed by OCT4, with the strongest staining for CD133 and NESTIN. In the non-cancerous tissue, as shown in Fig. 4, the immunoreactivities of NANOG (P<0.001) and OCT4 (P<0.001) were significantly greater than those of CD133 and NESTIN. In prostate cancer, NANOG (P<0.001) immunoreactivity was the strongest among the four stem cell markers (Fig. 5).

**Figure 6:**
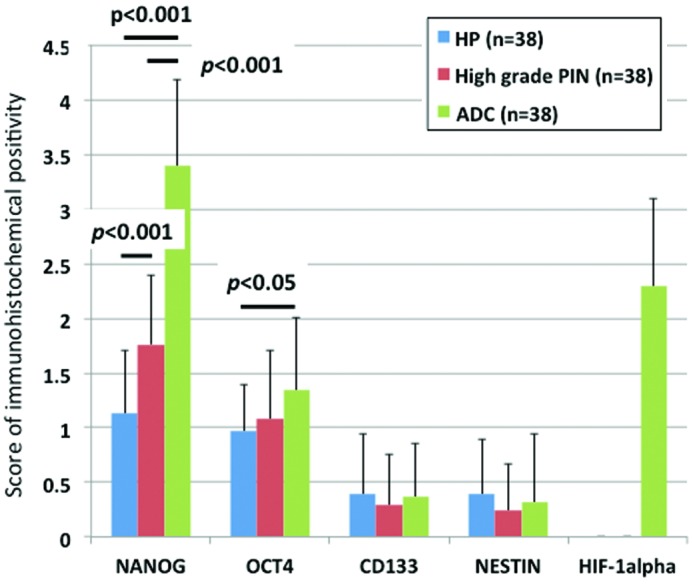
Immunohistochemical scores of four stem cell markers and HIF-1α in the hyperplastic glands (benign prostate hyperplasia), high-grade PIN and prostatic ADC. The score of NANOG in ADC was significantly greater than that of the hyperplastic glands (P<0.001) and high-grade PIN (P<0.001); the value of high-grade PIN was significantly higher than that of hyperplastic glands (P<0.001). The score of OCT4 in ADC was significantly higher than that in the hyperplastic glands (P<0.05), while the scores of CD133 and NESTIN of three lesions (hyperplastic glands, high-grade PIN and ADC) were almost similar. HIF-1α was expressed in the nuclei of ADC, but not in the hyperplastic glands and high-grade PIN. HP, hyperplastic; ADC, adenocarcinoma; HIF-1α, hypoxia-inducible factor-1α; PIN, prostate intraepithelial neoplasia; OCT4, octamer 4; CD133, cluster of differentiation 133.

**Figure 7:**
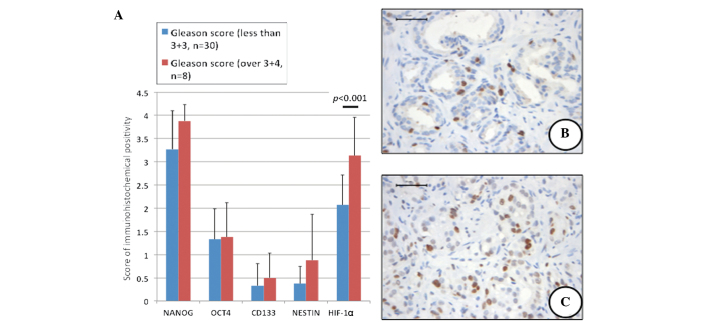
(A) Immunohistochemical scores of four stem cell markers and HIF-1α in prostatic ADC with Gleason scores of <3+3 and >3+4. No significant differences were identified between the scores of NANOG, OCT4, CD133 and NESTIN in the two different Gleason score groups. However, the score of HIF-1α was greater in ADC with Gleason scores of >3+4 than that in ADC with Gleason scores of <3+3 (P<0.001). The number of NANOG-positive cancer cells in (B) high Gleason score groups (3+4) was greater than that of (C) low Gleason score groups (2+2) (magnification, 200; scale bars, 50 μm). ADC, adenocarcinoma; HIF-1α, hypoxia-inducible factor-1α; OCT4, octamer 4; CD133, cluster of differentiation 133.

The expression score for NANOG in the prostate cancer cells was significantly greater than that of cells in high-grade PIN and the hyperplastic glands (P<0.001 for each comparison; Fig. 6). The number of atypical cells in high-grade PIN was also higher than that in the hyperplastic glands (P<0.001; Fig. 6). The expression of NANOG, OCT4, CD133 and NESTIN in prostate ADCs with high Gleason scores (>3+4) was greater than that in prostate cancers with low Gleason scores (<3+3), although this difference was not significant (Fig. 7).

**Figure 8:**
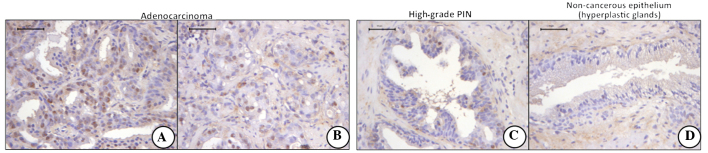
HIF-1α immunohistochemistry in ADC, high-grade PIN and hyperplastic glands. The number of HIF-1α-positive nuclei of (A and B) ADCs was greater than in (C) high-grade PIN. The number of HIF-1α-positive nuclei of (B) ADCs with Gleason scores of >3+4 was larger than that of (A) ADCs with Gleason scores of <3+3. No positive cell nuclei for HIF-1α were identified in the hyperplastic glandular cells (magnification, ×200; scale bars, 50 μm). ADC, adenocarcinoma; HIF-1α, hypoxia-inducible factor-1α.; PIN, prostate intraepithelial neoplasia.

HIF-1α immunohistochemistry revealed that specific cancer cell nuclei (Fig. 8A and B), corresponding to their Gleason score, as well as a few cell nuclei in high-grade PIN showed a positive reaction for HIF-1α (Fig. 8C). However, hyperplastic and normal glandular cells were negative for HIF-1α (Fig. 8D). The mean score for HIF-1α with a high Gleason score was significantly greater than that of HIF-1α with a low Gleason score (P<0.001; Fig. 7).

## Discussion

Pluripotency-associated transcription factors, including NANOG, Sox2 and OCT4, are known as regulators of cellular identity in ES cells (36) and have recently been identified in the epithelial malignancies of a variety of tissues (33,37), including prostate cancer (13,14,18). CD133 (23) and NESTIN (26) have also been reported to be expressed in prostate cancer. However, these reports were predominantly from human prostate cancer cell lines. Consistent with their role in sustaining the stemness of ES cells, pluripotency-related factors have been suggested to be expressed at a higher frequency in cancer exhibiting lower degrees of differentiation (37). In this study, the immunohistochemical expression of NANOG was markedly higher than that of the other stem cell markers, OCT4, CD133 and NESTIN. The reason for the discrepancy between the findings of the current study and those reported by others is not known; however, differences between prostate cancer obtained from biopsy specimens and human prostate cancer cell lines may have influenced the stainability of the four different stem cell markers, NANOG, OCT4, CD133 and NESTIN. Although Miki *et al* (8) observed tumor compartments and high-grade PIN with higher CD133 and an inverse correlation with androgen receptor staining, a CD133-positive reaction was not detected in the prostate cancer cells, PIN or hyperplastic glandular cells in this study.

CSCs comprise of ~0.01% of the tumor cell population. In this study, a large number of strongly positive NANOG and/or OCT4 cancer cells were observed. This high level of expression is not necessarily associated with stem cell behavior, but rather to the deregulation proteins that provide some type of growth advantage to cancer cells (38–41).

Androgen deprivation-induced atrophy of the prostate gland and subsequent regeneration following androgen replacement have indicated that the stem cell population may reside in the adult prostate gland in rodents (42). The origin of prostate cancer remains unknown and has given rise to a series of hypotheses (43). Prostate ADCs are frequently multifocal, show the same immunohistochemical profile as benign glandular cells, and lack basal cell markers, such as p63 and cytokeratin 34β. This indicates that prostate cancer may develop from altered benign glandular cells. However, multiple pluripotency markers, such as CD44, CD117 and Oct3/4, have been shown to be expressed in prostate cancer, indicating that prostate cancer may develop from common stem cell-like or intermediate cells (8,44). The findings on the expression of NANOG described in the current study may also support this hypothesis.

NANOG (9,10) is one of the four factors known to reprogram adult cells into germline-competent induced pluripotent stem cells (45). NANOG is also critical in maintaining the self-renewal and pluripotency of ES cells by regulating the cell fate of the pluripotent inner cell mass (46–48). Notably, elevated NANOG protein expression in several types of human cancer has been reported, predominantly in germ cell tumors, as well as the malignancies of non-germ cells (38), suggesting the involvement of NANOG in tumorigenesis and progression. Non-germ cell tumors, including breast (38) and oral cancer (49), also express NANOG. A systematic study using animal models and *in vitro* cell systems has provided substantial evidence for the key function of NANOG in human tumor development (50). A recent study has shown that the transforming growth factor (TGF)-β pathway is involved in the regulation of NANOG gene expression via binding with the NANOG proximal promoter (51). TGF-β functions as a key tumor suppressor of the prostate and can also promote malignant progression and metastasis of the advanced disease (52). Human cultured prostate cancer cells, prostate cancer xenografts and primary prostate cancer cells express a functional variant of NANOG, NANOG mRNA, in cancer cells (50). This expression is derived predominantly from a retrogene locus termed NANOGp8 (50). In this study, the NANOG protein was detected in the nucleus of cancer cells, but was not expressed in hyperplastic glandular cells. These findings suggest that NANOG has a particular function in prostate cancer development. In addition, a significant correlation has been reported between NANOG-, OCT4- and HIF-1α-positive regions (31). Low oxygen levels promote self-renewal in stem cells and hypoxia has been associated with an aggressive disease course and poor clinical outcomes in malignancies, including prostate cancer (28,29). Furthermore, a number of aggressive neoplasms exhibit gene expression signatures characteristic of human ES cells. Thus, HIF may act as a key inducer of a dynamic state of stemness in pathological conditions.

OCT4 maintains pluripotency in embryogenesis; the upregulation of OCT4 results in differentiation to the primitive endoderm and mesoderm, while downregulation induces a loss of pluripotency and dedifferentiation into the trophectoderm (53). A recent report questioned the function of OCT4 as a pure stem cell marker by showing its expression in differentiated cells (54). Ugolkov *et al* (18) reported that OCT4 nuclear expression was markedly associated with benign prostatic lesions, but not prostate cancer. In the present study, OCT4 expression was found in the prostate cancer and non-cancerous glandular cells; however, differences were observed in its expression between prostate cancer and non-cancerous glands. Although, these differences were not as marked as those observed in NANOG expression.

One notable observation in the current study was that prostate cancer cells expressing NANOG and OCT4 were also positive for HIF-1α reactivity. Hypoxia-regulated genes are mediated by the HIF-1 complex composed of a heterodimeric pair of HIF-1α and -1β (28,29), and HIF-1α is an important transcription factor in prostate carcinogenesis, which suggests that HIF-1α may be a potential prognostic biomarker in the proteomic assessments of prostate cancers (55,56). Additionally, HIF-1α induces human ES cell markers, such as NANOG (14,30,31), OCT4 (14,30,31) and CD133 (32), in cancer cells. The findings of the current study showing the coexpression of NANOG, OCT4 and HIF-1α support these studies. However, a slightly positive reaction or null of CD133 in cancer cells was observed. The reason for this was unknown; although, a strong correlation was identified between NANOG and HIF-1α expression, which may suggest that NANOG and HIF-1α co-operate in prostate carcinogenesis.

The results of this study showed that of the four CSC markers examined (NANOG, OCT4, CD133 and NESTIN), NANOG was intensively expressed in prostate cancer. In addition, HIF-1α was coexpressed in cancer cells. These findings suggest that NANOG, in conjunction with HIF-1α, may be important in prostate carcinogenesis. In addition, the immunohistochemical expression of NANOG may present as a biomarker for investigating the pathobiology of prostate cancer.
